# Electrocardiograms Revealing Epsilon Waves Following Use of Hormone Supplements in Young Cardiac Arrest Patient

**DOI:** 10.7759/cureus.14305

**Published:** 2021-04-05

**Authors:** Enola R Okonkwo, Christian Schuetz, Bryan Hyman, Brian Samuels, Dany Sayad, James Bower

**Affiliations:** 1 Emergency Medicine, University of South Florida Morsani College of Medicine, Tampa, USA; 2 Internal Medicine, University of South Florida Morsani College of Medicine, Tampa, USA; 3 Cardiology, Sanger Heart & Vascular Institute, Charlotte, USA

**Keywords:** hyperthyroidism, exogenous steroids, arrhythmogenic cardiomyopathy, arrhythmia, thyroid supplementation, epsilon wave, sudden cardiac death

## Abstract

Introduction: An underlying cardiomyopathy should be suspected in young patients presenting with ventricular arrhythmias and sudden cardiac arrest. Electrocardiograms revealing epsilon waves are associated with many serious conditions such as arrhythmogenic right ventricular cardiomyopathy, posterior myocardial infarction, right ventricular infarction, infiltration disease, sarcoidosis, Brugada Syndrome, Tetralogy of Fallot, and hypothermia. This case report features epsilon waves in a young cardiac arrest patient suspected of having an unrecognized cardiomyopathy that resulted in a fatal arrhythmia in the setting of exogenous bovine thyroid hormone and steroid use.

Case presentation: A previously healthy 33-year-old male with a history of anabolic steroid use and bovine thyroid hormone use presented to the emergency department following witnessed cardiac arrest with bystander cardiopulmonary resuscitation (CPR). Upon emergency medical service (EMS) arrival, the patient was in ventricular fibrillation and received defibrillation with the return of spontaneous circulation. In the emergency department, he was unresponsive and required norepinephrine to maintain blood pressure. An epsilon wave and a prolonged QTc interval were noted on his electrocardiogram (ECG). CT angiogram of the chest and CT head were negative for acute abnormalities. Pertinent laboratory work-up included a lactate level of 12.0 mmol/L, thyroid-stimulating hormone of 0.02 ulU/L, and a free thyroxine level of 0.04 ng/dL. Cardiac ultrasound showed globally decreasedleft ventricular function with an ejection fraction of 25-30% and mild dilation of the right ventricle. A cardiac MRI was ordered but the patient had recurrent ventricular fibrillation and was too unstable to complete. He suffered anoxic brain injury with no improvements in neurologic function and was transitioned to comfort care. The patient died two months later in hospice care. The cause of cardiac arrest was attributed to the patient’s steroid and bovine thyroid supplementation, but autopsy results revealed histologic evidence of possible arrhythmogenic right ventricular cardiomyopathy.

Discussion: Epsilon waves are widely known to be associated with structural abnormalities of the heart, most notably, arrhythmogenic right ventricular cardiomyopathies. Epsilon waves may be present in a variety of other medical conditions including posterior myocardial infarction, right ventricular infarction, infiltration disease, sarcoidosis, Brugada Syndrome, Tetralogy of Fallot, and hypothermia. This case report describes an epsilon wave found in a patient with suspected arrhythmogenic right ventricular cardiomyopathy that suffered a fatal arrhythmia triggered by bovine thyroid hormone and steroid use.

## Introduction

An underlying cardiomyopathy should be suspected in young patients presenting with ventricular arrhythmias and sudden cardiac arrest. Electrocardiograms revealing epsilon waves are associated with many serious conditions such as arrhythmogenic right ventricular cardiomyopathy, posterior myocardial infarction, right ventricular infarction, infiltration disease, sarcoidosis, Brugada Syndrome, Tetralogy of Fallot, and hypothermia. This case report features epsilon waves in a young cardiac arrest patient suspected of having an unrecognized cardiomyopathy that resulted in a fatal arrhythmia in the setting of exogenous bovine thyroid hormone and steroid use. 

## Case presentation

A previously healthy 33-year-old male presented to the emergency department after suffering from a cardiac arrest at home. According to his family, he had been in his normal state of health until one week prior when he reported fatigue. The patient was a bodybuilder and had a history of prolonged anabolic steroid use. He recently started using bovine levothyroxine not intended for human consumption to enhance his performance. On the day of presentation, the patient began experiencing difficulty breathing, became unresponsive, and developed cardiopulmonary arrest followed immediately by bystander cardiopulmonary resuscitation (CPR). 

**Figure 1:**
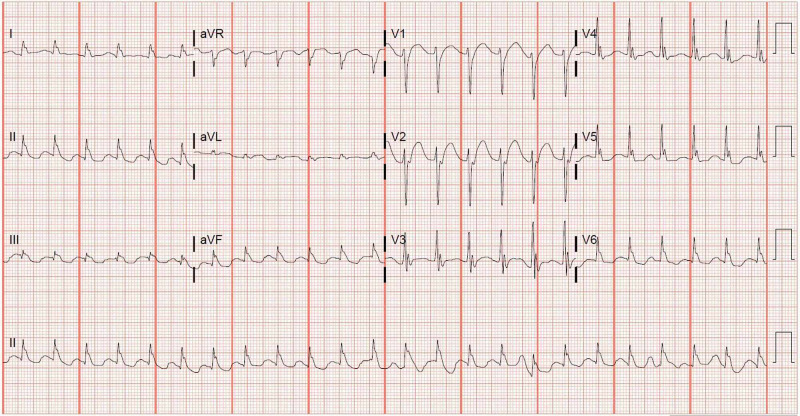
Initial electrocardiogram obtained in emergency department

Upon emergency medical services (EMS) arrival, the patient was found to be in ventricular fibrillation (VF) and was defibrillated. CPR was continued and he received two rounds of 1 mg epinephrine and 2 mg total of Narcan® (Emergent BioSolutions, Plymouth Meeting, PA, USA) with a return of spontaneous circulation (ROSC). A laryngeal mask airway was placed by EMS. On arrival to the emergency department, the patient remained unresponsive with an initial heart rate of 140, blood pressure of 90/60 mmHg, respirations via assisted ventilation at a rate of 12, and temperature of 36.4°C. He was started on norepinephrine at a rate of 0.05 mcg/kg/min with improvement in blood pressure to 115/71. His initial ECG (Figure 1) revealed tachycardia with ε waves in V1-V3, prolonged QRS interval of 141 ms, and a prolonged QTc interval of 597 ms. 

Following the ECG, he received 100 meq of sodium bicarbonate, 2 grams of magnesium, and 1 gram of calcium chloride. His neurological examination showed fixed and dilated pupils and decerebrate posturing. A definitive airway was then placed with a 7.5 endotracheal tube. The patient received 20 mg of etomidate and 100 mg of rocuronium during intubation and was started on a propofol drip following the procedure. CT angiogram of the chest was negative for pulmonary embolism and CT head was negative for acute findings. Pertinent laboratory workup in the emergency department (Table 1) included a lactate level of 12.0 mmol/L, thyroid-stimulating hormone of 0.02 uIU/L, and a free thyroxine level of 0.04 ng/dL. He was admitted to the cardiac intensive care unit for further workup.

**Table 1:** Laboratory results in the emergency department Blood gas analysis represents an arterial sample.  Drug screen analysis performed on urine sample.  Abbreviations: pO2: pressure of oxygen, pCO2: pressure of carbon dioxide, SO2: saturation  of oxygen, CoHb: carboxyhemoglobin, HCO3: bicarbonate concentration, Glu: glucose, Cre: creatinine, Na: sodium, K: potassium, Cl: chloride, Ca: calcium, ALT: alanine aminotransferase, AST: aspartate aminotransferase, WBC: white blood cell, Hg: hemoglobin, Plt: platelet, TSH: thyroid-stimulating hormone, free T4: free thyroxine, free T3: free triiodothyronine, total T4: total thyroxine, total T3: total triiodothyronine

	Result	Reference range
pH	7.22	7.35 - 7.45
pO_2_	536	75 - 110 MM HG
PCO_2_	44	35 - 45 MM HG
SO_2_%	98	>95 %
CoHb	0.3	0 - 3 %
HCO_3_	18	24 - 27 MEQ/L
Anion gap	21	5 - 13 MEQ/L
Base deficit	9	0 - 2 MEQ/L
Glu	163	70 - 110 MG/DL
Cre	1.5	0.72 - 1.25 MG/DL
Na	139	135 - 148 MEQ/L
K	4.3	3.5 - 5.3 MEQ/L
Cl	105	98 - 107 MEQ/L
Ca	9.3	8.5 - 10.5 MG/DL
ALT	570	5.0 - 55 U/L
AST	421	5.0 - 34 U/L
WBC	10.6	4.6 - 10.2 10*3/uL
Hg	15.9	14.1 - 18.1 g/dL
Plt	146	142.0 - 424.0 10*3/uL
Lactate	12.0	0.5 - 2.2 MMOL/L
TSH	0.02	0.35 - 4.94 UIU/ML
Free T4	0.40	0.70 - 1.48 NG/DL
Free T3	1.63	1.71 - 3.71 PG/ML
Total T4	0.91	4.87 - 11.72 UG/DL
Total T3	0.33	0.58 - 1.59 NG/ML
Troponin	0.017	0.000 - 0.028 NG/ML
salicylate	<5.0	2.8 - 20.0 MG/DL
acetaminophen	<3.0	10 - 20 UG/ML
amphetamine	negative	negative
cocaine	negative	negative
benzodiazepines	negative	negative
cannabinoids	negative	negative
barbiturates	negative	negative
phencyclidine	negative	negative
ethanol	<10	<10 MG/DL
methadone	negative	negative
oxycodone	negative	negative

**Figure 2:**
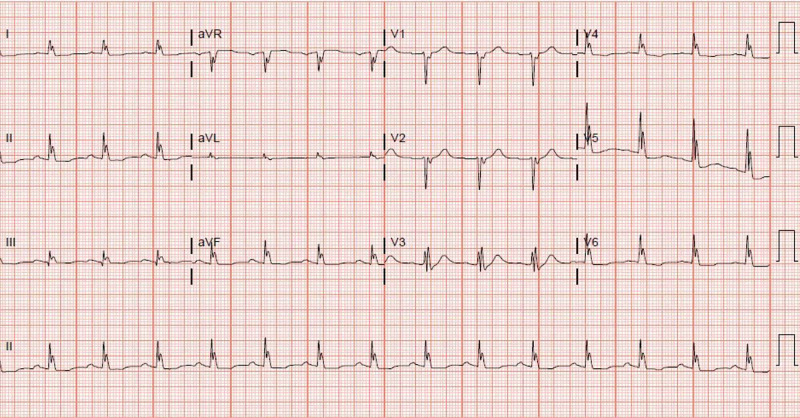
Electrocardiogram obtained in the cardiac intensive care unit following recurrent VF arrest

Upon arrival to the cardiac unit, the patient experienced recurrent VF. He underwent three rounds of advanced cardiac life support (ACLS) receiving immediate defibrillation followed by 1 mg epinephrine, 300 mg amiodarone, a second dose of 150 mg amiodarone, 50 meq of sodium bicarbonate, and 3 grams of magnesium before achieving ROSC. Post ROSC, ECG again demonstrated epsilon waves and diffuse conductive abnormalities (Figure 2). The patient's temperature at the time of ECG was 35.6°C. His recurrent VF arrest was thought to be related to the R-on-T phenomenon. He was started on lidocaine infusion at 0.5 mg/min and an isoproterenol infusion at 0.2 mcg/min. Therapeutic hypothermia protocol was initiated following his recurrent VF arrest. Formal cardiac echocardiogram revealed diffuse hypokinesis with a left ventricular ejection fraction of 25-30% and mild right ventricular dilation. A cardiac MRI was ordered but ultimately was unable to be obtained secondary to the patient’s critical condition. 

The patient completed rewarming from the hypothermia protocol three days after admission but remained comatose with no neurologic recovery. Electroencephalogram revealed nonconvulsive status epilepticus and MRI demonstrated diffuse anoxic brain injury with poor prognosis. Upon further consultation with neurology and palliative care, the family decided to pursue comfort measures only. Once hospice placement was finalized, the patient was extubated and transferred out of the hospital. The patient died two months later. An autopsy was performed and his cause of death was reported as anoxic encephalopathy following resuscitated cardiopulmonary arrest due to apparent exogenous thyroid toxicity. No chronic dilation was found in the chambers of the heart. The right ventricle (RV) histologic slide demonstrated a focus of mature adipocytes and adjacent fibrous tissue with a paucity of myocytes. 

## Discussion

ECGs can quickly provide valuable diagnostic information in emergent situations. Epsilon waves are characterized as abnormal depolarizations occurring between the end of a QRS complex and the beginning of a T wave. The cause of this unusual electrical signal is due to the impeded conduction within the ventricular myocardium, inducing post-excitation of the myocytes in the ventricle. The ε waves are usually noted in the right precordial leads V1-V3 but can also present in left precordial leads with left-sided cardiomyopathies [1]. The degree of ECG abnormalities appears to correlate with disease severity and arrhythmogenic risks [2,3]. Given the low amplitude nature of ε waves, a minimum setting of 150 Hz is required for proper observation between the QRS complex and T wave [1,4]. Without this proper setting, ECGs may be unable to detect these low voltage signals, leading to possible misdiagnosis or delays in diagnosis and treatments. The use of Fontaine leads can further increase the sensitivity of detecting epsilon waves [5].

Epsilon waves are widely known to be associated with structural abnormalities of the heart, most notably arrhythmogenic right ventricular cardiomyopathies (ARVCs). The term ARVCs was introduced before the First International Symposium in Paris in 1996 and encompasses multiple diseases affecting the conduction of the right heart such as right ventricular outflow tract ventricular tachycardia (VT), Brugada syndrome, and arrhythmogenic right ventricular dysplasia (ARVD) [3]. ARVD as described by Marcus et al. [6] remains the most notable in emergency medicine secondary to its association with ventricular arrhythmias. ARVD is now recognized as the second most common cause of sudden cardiac death in young people, causing up to 20% of sudden cardiac deaths in patients under 35 years of age [7]. A variety of other medical conditions such as posterior myocardial infarction, right ventricular infarction, infiltration disease, sarcoidosis, Brugada Syndrome, Tetralogy of Fallot, and hypothermia are known to demonstrate ε waves [1,8-11]. This case report likely represents a patient who had an unrecognized underlying cardiomyopathy, characterized by fibrofatty infiltrates on cardiac biopsy, which predisposed the patient to arrhythmia. The addition of toxicologic agents such as bovine thyroid hormone and steriod use likely further contributed to the patient's risk of arrhythmia. 

Literature review failed to identify prior reports of steroids or thyroid supplements inducing ε waves but numerous cardiac adverse effects have been reported. It is widely known that steroid supplements may induce cardiomyopathies most commonly characterized by left ventricular hypertrophy and dilation of cardiac chambers leading to associated risks of arrhythmias and even cardiovascular-related death [12-15]. Bovine thyroid supplementation has also been reported to induce arrhythmia. In one case report, a patient presented to the ED with chest tightness and palpitations after taking “natural” bovine thyroid supplements in lieu of levothyroxine for her hypothyroidism [16]. ECG results at the time confirmed atrial fibrillation with rapid ventricular response, a common arrhythmia seen in thyrotoxicosis, but did not show ε waves. Thyrotoxicosis is a recognized serious health condition that can lead to arrhythmias, heart failure, and lethal outcomes if proper medical attention is not sought [16-17]. 

The etiology of the patient's suspected underlying cardiomyopathy is unclear. His long-term use of anabolic steroids may have contributed to cardiomyopathy. The histologic autopsy findings which demonstrated a paucity of RV myocardial fibers and an increase in fatty infiltration are consistent with ARVD [6, 18] but his autopsy failed to reveal any overt enlargement of the RV which has been reported as a common feature of ARVD [6]. His ECG also lacked the usual T wave inversions found in ARVD [6, 19]. His echocardiogram showed clearly abnormal cardiac function with a left ventricular ejection fraction of 25-30% and mild right ventricular dilation. It is difficult to interpret the significance of these findings in the setting of a recent cardiac arrest. Despite these inconsistencies, it is important to note that patients with ARVC experience progressive disease and are prone to arrhythmias out of proportion to the structural changes present when compared to other forms of cardiomyopathy [18]. 50% of ARVC patients are found to have an inherited disease pattern related to mutations in desmosomal proteins, though recessive and unexplained cases have been reported [18]. Our patient had no family history of ARVC and no genetic testing was performed. 

It is also important to address the similarities between the ε wave and the J wave associated with hypothermia, especially in the setting of cardiac arrest. The J wave, also known as the Osborn wave, is a positive deflection at the terminal part of the QRS seen in hypothermia [20]. This ECG finding looks very similar to the ε wave. Hypothermia is important to consider in any patient presenting with ε waves. The major differentiating factor is the presence of hypothermia. Our patient's temperature at the time of his initial ECG was 36.4°C. A 2013 study [20] involving hypothermic patients found no hypothermia-induced J waves in patients with a temperature > 33.5°C. J waves were more commonly seen in patients presenting with severe hypothermia [20]. Our patient was normothermic, ruling out the possibility of hypothermic-induced ECG abnormalities. 

The possibility of congenital and acquired long QTc, channelopathies, and other toxicologic etiologies such as sodium channel blocker toxicity, must also be considered in this case given the abnormal QTc and QRS durations. The patient had no reported family history and no other known ingestions. 

## Conclusions

The ε wave has been noted in a variety of medical conditions such as arrhythmogenic right ventricular cardiomyopathy, posterior myocardial infarction, right ventricular infarction, infiltration disease, sarcoidosis, Brugada Syndrome, Tetralogy of Fallot, and hypothermia. We were presented with an unusual case in which exogenous hormone supplements may have contributed to cardiac arrest in a young patient noted to have epsilon waves on ECG. Based on the patient's ECG, echocardiogram, and autopsy findings, this patient likely had an unrecognized cardiomyopathy such as ARVC, which predisposed him to an arrhythmia. The use of exogenous bovine thyroid supplement and steroids further lowered his arrhythmia threshold. 

Regardless of the etiology of the epsilon wave, physicians should be aware of this ECG finding and the increased likelihood for fatal arrhythmias in patients demonstrating this abnormality. It is especially important for physicians to look for the presence of an epsilon wave in patients presenting to the emergency department for evaluation of cardiac arrest, syncope, palpitations, dyspnea, chest pain, and seizure. If epsilon waves are identified, patients may be able to receive antiarrhythmic medications or an implantable cardioverter-defibrillator to decrease the chance of sudden cardiac death. 
